# From Foundational Caregiving to Maternal Eudaimonic Well-Being: The Role of Parenting and Interaction Competence

**DOI:** 10.3390/healthcare14152377

**Published:** 2026-08-04

**Authors:** Jo-Lin Chen, Shu-Ling Cho, Shou-Chi Huang, Yen-Cheng Chen

**Affiliations:** 1 Department of Child and Family Studies, Fu Jen Catholic University, New Taipei City 242062, Taiwan; 046286@mail.fju.edu.tw (J.-L.C.); 142634@mail.fju.edu.tw (S.-C.H.); 2 Department of Clinical Psychology, Fu Jen Catholic University, New Taipei City 242062, Taiwan; lincho056384@gmail.com; 3 Department of Family Science, Chinese Culture University, Taipei 11114, Taiwan

**Keywords:** developmental parenting and interaction competence, early parenthood, eudaimonic parental well-being, first-time mothers, foundational caregiving competence, maternal mental health

## Abstract

**Background/Objectives:** The transition to early parenthood is a pivotal phase influencing maternal mental health. Although parenting competence may support maternal adjustment, it is often treated as a unitary construct. This study examined the associations among foundational caregiving competence, developmental parenting and interaction competence, and eudaimonic parental well-being in first-time mothers. **Methods:** A cross-sectional survey was conducted with 372 first-time mothers whose young children attended licensed infant daycare centers in northern Taiwan. Participants completed questionnaires regarding the three study constructs and demographic characteristics. Data were analyzed using descriptive statistics, Pearson correlations, and partial least squares structural equation modeling. **Results:** Foundational caregiving competence was strongly associated with developmental parenting and interaction competence, which was positively associated with eudaimonic parental well-being. The direct association between foundational caregiving competence and eudaimonic parental well-being was not statistically significant after developmental parenting and interaction competence was included in the model. However, a significant indirect association was observed through developmental parenting and interaction competence. **Conclusions:** Relational, developmental, and role-oriented parenting competence was more closely associated with mothers’ experiences of meaning, fulfillment, and relational satisfaction than practical caregiving competence alone. Given the cross-sectional design and modest explanatory power, the findings represent preliminary associations rather than causal evidence. They may inform future parenting-support services that distinguish practical caregiving capacities from relational and developmental parenting competencies.

## 1. Introduction

The transition to early parenthood constitutes a critical period for women’s mental health. First-time mothers must adapt to new caregiving responsibilities, changing family roles, and intensified emotional demands. Deave et al. [1] showed that parents often require substantial informational and emotional support during pregnancy and early parenthood, while Lorén et al. [2] emphasized that the transition to motherhood is shaped by both individual and environmental conditions. During this period, maternal well-being cannot be understood solely through the presence or absence of psychological distress. It also involves the psychosocial resources through which women interpret caregiving demands, sustain parental confidence, and negotiate relational adjustment within the parenting role [3,4].

Research on early parenthood has increasingly demonstrated that parenting stress, psychological distress, and relational adaptation are closely intertwined. Babore et al. [5] found that parenting stress plays an important role in the mental health of mothers and children. Fang et al. [6] identified parent-related, child-related, and situational factors as key contributors to parenting stress, while Rusu et al. [7] reported that parental stress is systematically associated with lower well-being. In Taiwan, Huang et al. [8] further showed that maternal confidence and social support are closely related to parenting stress among first-time mothers, underscoring the need to examine maternal adaptation within culturally situated contexts. Mothers may also encounter strong expectations regarding competent caregiving, emotional availability, responsibility for children’s development, and the maintenance of family harmony. Such expectations may shape how mothers evaluate their parenting competence and psychological adjustment. This context therefore provides a meaningful setting in which to examine how different domains of parenting competence are associated with positive maternal well-being.

Parenting competence represents an important psychosocial resource through which mothers may negotiate the demands of early parenthood. Bandura’s self-efficacy framework provides a useful foundation for understanding competence-related beliefs, as perceived capability influences how individuals appraise challenges, organize action, regulate effort, and persist under demanding conditions [9]. Parenting self-efficacy is domain-specific rather than global, meaning that parents’ perceived competence may vary across different functional domains. Mothers may therefore feel confident in practical caregiving while reporting different levels of confidence in relational, developmental, or role-management aspects of parenting. In parenting research, perceived parenting competence and parenting self-efficacy have been associated with parents’ confidence in caregiving and their perceived ability to meet children’s needs [10,11]. Empirical studies have also linked parenting competence and early postnatal caregiving confidence with maternal adjustment [12,13]. However, much of this research has treated parenting competence as a broad or global construct, potentially obscuring meaningful differences among the functional domains of early parenting.

Recent scholarship suggests that parental competencies should be conceptualized as multidimensional capacities rather than as a generalized sense of efficacy. Lorence et al. [14] argued that parental competencies involve multiple capacities embedded within specific parenting contexts. Talebi et al. [15] similarly found that parenting preparation is associated with maternal role competence, indicating that competence may vary across domains of parenting practice. Building on this perspective, the present study distinguishes foundational caregiving competence from developmental parenting and interaction competence. This distinction is also consistent with Taiwanese family education research, in which parenting capacity has been conceptualized as a learnable and contextually situated set of competencies [16,17].

The distinction between these two competence domains is theoretically important. Foundational caregiving competence reflects mothers’ perceived capacity to manage the practical and protective demands of childrearing, including routine care, developmental knowledge, health, safety, and protection. Developmental parenting and interaction competence, by contrast, reflects the relational, developmental, and role-oriented capacities through which parenting is enacted in daily family life, including emotional responsiveness, behavioral guidance, parent–child interaction and shared learning, parental role adaptation, and family management. The former is primarily practical and protective in orientation, whereas the latter concerns the relational enactment and broader organization of parenting. While closely interrelated, these domains constitute functionally distinct facets of perceived capability, rather than merely interchangeable manifestations of a generalized parenting efficacy.

Eudaimonic parental well-being offers a suitable framework for examining the positive psychological dimensions of early motherhood. Diener [18] conceptualized subjective well-being in terms of individuals’ evaluative and affective experiences, whereas Keyes [19] extended the discussion of mental health by emphasizing positive functioning, meaning, and flourishing. Park et al. [20] further argued that emotional well-being should be understood in relation to broader experiences of functioning and life engagement. In the parenting context, well-being is not limited to reduced stress or positive affect. It also includes the meaning, achievement, and fulfillment that parents derive from the parenting role [21,22]. Chen and Lee [23] similarly described parenting joy in Taiwanese families as a positive experience grounded in parental meaning, parent–child closeness, and witnessing children’s growth.

In the present study, eudaimonic parental well-being was conceptualized as a positive psychological outcome rather than as another domain of parenting competence. This positioning is consistent with the view that well-being involves positive functioning, meaning, and life engagement rather than only positive affect or the absence of distress [19,20]. Developmental parenting and interaction competence concerns mothers’ perceived capacity to enact responsiveness, behavioral guidance, shared learning, and role management, whereas eudaimonic parental well-being concerns the psychological meanings mothers derive from parenting, including fulfillment, achievement, and relational satisfaction within the parenting role [21,22]. Although parent–child closeness was conceptualized as one dimension of the outcome construct, it captured mothers’ perceived relational satisfaction rather than evaluating behavioral parenting practices [23]. Accordingly, the model distinguishes between perceived capability for parenting practice and the positive psychological experience derived from the parenting role.

Consistent with Bandura’s self-efficacy framework, mastery experiences in concrete and recurring caregiving tasks are posited to bolster mothers’ perceived capability to manage broader parenting demands [9]. Consequently, foundational caregiving competence may support mothers’ competence in more relationally and contextually complex parenting practices [10,14]. Developmental parenting and interaction competence may, in turn, be more closely associated with the psychological rewards that mothers derive from parenting, including meaning, fulfillment, achievement, and relational satisfaction [21,22]. This hierarchical ordering represents a theoretically informed functional specification rather than an empirically established temporal sequence. Given the cross-sectional design, these relationships are interpreted as statistical associations rather than causal or temporal relationships.

Despite increasing attention to maternal mental health, parenting stress, and support during early parenthood, existing research has often treated parenting competence as a broad construct. Middleton et al. [24] and Refaeli et al. [25] emphasized the importance of support during the transition to parenthood, yet less attention has been given to whether different parenting competence domains occupy distinct functional positions in relation to maternal well-being. The present study therefore does not simply examine whether parenting competence is associated with well-being. Rather, it investigates whether foundational caregiving competence and developmental parenting and interaction competence are differentially associated with eudaimonic parental well-being among first-time mothers.

Accordingly, this study examined the associations among the three constructs in a sample of first-time mothers in Taiwan. Foundational caregiving competence was specified as the predictor, developmental parenting and interaction competence as the intermediary construct, and eudaimonic parental well-being as the outcome. The model was designed to clarify whether foundational caregiving competence is directly associated with eudaimonic parental well-being and whether a significant indirect association is observed through developmental parenting and interaction competence. Given the cross-sectional design, the model was not intended to establish causal or temporal ordering. By differentiating the functional positions of two parenting competence domains, this study provides preliminary evidence regarding their potentially distinct associations with eudaimonic parental well-being during early parenthood [3,8,25].

Based on this conceptual rationale, the following hypotheses were tested:

**H1.**  *Foundational caregiving competence is positively associated with developmental parenting and interaction competence among first-time mothers.*

**H2.**  *Developmental parenting and interaction competence is positively associated with eudaimonic parental well-being among first-time mothers.*

**H3.**  *Foundational caregiving competence has a positive indirect association with eudaimonic parental well-being through developmental parenting and interaction competence.*

## 2. Materials and Methods

### 2.1. Study Design

This study employed a cross-sectional survey design to examine the associations among foundational caregiving competence, developmental parenting and interaction competence, and eudaimonic parental well-being among first-time mothers during early childrearing. Developmental parenting and interaction competence was examined as an intermediary construct in the indirect association between foundational caregiving competence and eudaimonic parental well-being. Given the cross-sectional nature of the data, developmental parenting and interaction competence was treated as an intermediary construct within an associative model, without implying causal relationships or temporal ordering.

Data were collected using a structured questionnaire administered to first-time mothers whose young children were enrolled in licensed infant daycare centers in northern Taiwan. The questionnaire assessed mothers’ perceived foundational caregiving competence, developmental parenting and interaction competence, and eudaimonic parental well-being. This design enabled the simultaneous examination of the associations among these constructs within the context of early parenthood.

The research protocol was approved by the Institutional Review Board of Fu Jen Catholic University (IRB No. C107169). The Institutional Review Board waived the requirement for informed consent because the study involved an anonymous questionnaire, collected no personal identification information, and posed minimal risk to participants. Participation was voluntary, and all study procedures were conducted in accordance with the approved ethical protocol. The data were collected, stored, and analyzed anonymously.

### 2.2. Participants and Data Collection

Participants comprised first-time mothers utilizing 17 licensed infant daycare centers in northern Taiwan. This cohort was specifically sampled to capture the intersection of transitioning into parenthood and early reliance on formal institutional care. Unlike mothers engaged in full-time maternal care or informal kinship care, these women are uniquely positioned to experience complex demands, including coordinating with childcare professionals, adjusting to maternal–child separation, and balancing family and employment responsibilities. Daycare enrollment served as the institutional context for recruitment rather than an explanatory variable.

To be eligible, participants had to be mothers raising their first child who was enrolled in one of the participating centers. No exclusion criteria were applied regarding multiple births, preterm delivery, child disability, or maternal mental health history; consequently, these characteristics were not assessed. Daycare staff served solely to distribute the study invitations, information sheets, and questionnaires.

Participation was entirely voluntary. Mothers could decline participation or return a blank questionnaire without any negative consequences, and their decision did not affect their access to childcare services. Questionnaires were completed anonymously, contained no identifying information, and were coded strictly for data management and statistical analysis purposes.

As a token of appreciation, participants received a small incentive. Of the 432 questionnaires distributed, 372 usable responses were obtained after excluding incomplete or invalid questionnaires, yielding a response rate of 86.11%.

### 2.3. Measures

The survey included demographic items and three measures assessing foundational caregiving competence, developmental parenting and interaction competence, and eudaimonic parental well-being. The parenting competence measures were derived from Taiwanese studies of adaptive parenting competence and parenting education needs [16,17]. Items were organized according to their conceptual relevance to practical caregiving, developmental knowledge, safety and protection, emotional responsiveness, behavioral guidance, parent–child interaction, parental role adaptation, and family management.

The eudaimonic parental well-being measure was adapted from Chen and Lee’s parental joy scale [23]. Items assessing meaning, achievement, fulfillment, and relational satisfaction were retained, whereas less relevant items were excluded. Minor wording adjustments were made to enhance clarity while preserving conceptual equivalence. The resulting measures were assessed for internal consistency, composite reliability, convergent validity, and discriminant validity.

Demographic information included maternal age and education, child gender, and date of birth. Child age in months was calculated from the reported date of birth.

#### 2.3.1. Foundational Caregiving Competence Scale

Foundational caregiving competence was assessed using a scale derived from a Taiwanese study of adaptive parenting competence among first-time parents [16]. The scale assessed perceived competence in practical caregiving, developmental knowledge, and child safety and protection.

The 21-item scale comprised childcare competence (9 items), young child development knowledge (7 items), and child safety and protection (5 items). Responses ranged from 1 (fully incompetent) to 5 (fully competent), with higher scores indicating greater foundational caregiving competence. Cronbach’s α values for the total scale and subscales ranged from 0.92 to 0.96, and the three dimensions explained 73.75% of the total variance.

#### 2.3.2. Developmental Parenting and Interaction Competence Scale

Developmental parenting and interaction competence was assessed using a scale derived from a Taiwanese study of parental capacities and parenting education needs [17]. The scale assessed perceived competence in parent–child interaction, emotional responsiveness, behavioral guidance, parental role adaptation, and family management.

The 21-item scale comprised parental role and family management (7 items), parental warmth and responsiveness (5 items), parent–child interaction and shared learning (4 items), and parental guidance and regulation (5 items). Responses ranged from 1 (fully incompetent) to 5 (fully competent), with higher scores indicating greater developmental parenting and interaction competence. Cronbach’s α values for the total scale and subscales ranged from 0.90 to 0.95, and the four dimensions explained 75.25% of the total variance.

#### 2.3.3. Eudaimonic Parental Well-Being Scale

Eudaimonic parental well-being was assessed using items adapted from the parental joy scale developed by Chen and Lee [23]. The original scale assessed positive parenting experiences in Taiwanese families, including meaning derived from the parenting role, achievement associated with children’s growth, and satisfaction arising from parent–child relationships.

Item selection was guided by the conceptual definition of eudaimonic parental well-being adopted in this study. Items were retained when they reflected meaning and fulfillment in parenting, achievement derived from children’s growth and development, or relational satisfaction and closeness. Items less directly aligned with these domains were excluded, and minor wording adjustments were made to improve clarity and relevance for first-time mothers with young children while preserving the original meaning.

The adapted items were reviewed by experts to evaluate their relevance, clarity, and conceptual consistency with eudaimonic parental well-being. The final scale comprised 20 items across three dimensions: meaning and fulfillment in parenting, parental sense of achievement from young children’s development, and parent–child closeness. Parent–child closeness reflected mothers’ perceived relational satisfaction rather than parenting behavior. Responses ranged from 1 (strongly disagree) to 5 (strongly agree), with higher scores indicating greater eudaimonic parental well-being. Cronbach’s α values were 0.82, 0.86, and 0.93 for the three subscales, respectively, and 0.94 for the overall scale. The adapted measure was further evaluated through composite reliability, convergent validity, and discriminant validity.

### 2.4. Data Analysis Strategy

Data were analyzed using IBM SPSS Statistics 29 and SmartPLS 3. Descriptive statistics summarized participant characteristics and study variables, Cronbach’s α assessed internal consistency, and Pearson correlations examined bivariate associations.

PLS-SEM was used to assess the measurement and structural models because the study emphasized explained variance and direct and indirect associations in a parsimonious model using contextually adapted measures [26]. Although covariance-based SEM was also a viable option for confirmatory testing, PLS-SEM was more consistent with the study’s variance-oriented objectives. The measurement model was evaluated using indicator loadings, Cronbach’s α, composite reliability, average variance extracted, and HTMT.

Foundational caregiving competence was specified as the exogenous predictor, developmental parenting and interaction competence as the intermediary construct, and eudaimonic parental well-being as the endogenous outcome. Structural paths and the indirect association were tested using 5000 bootstrap resamples at a significance level of 0.05. Multicollinearity was assessed using VIF values, with 3.3 as the conservative threshold [27]; VIF was treated only as an initial diagnostic of common method bias.

Effect sizes, predictive relevance, and out-of-sample predictive performance were assessed using *f*^2^, Stone–Geisser *Q*^2^, and PLSpredict RMSE comparisons with the linear benchmark model, respectively. Given the cross-sectional design, all paths were interpreted as statistical associations rather than causal or temporal relationships.

## 3. Results

### 3.1. Participant Characteristics

A total of 372 first-time mothers participated in this study. Their ages ranged from 22 to 45 years, with a mean age of 34.34 years. Most participants had completed university-level education (*n* = 261, 70.2%), followed by graduate-level education (*n* = 65, 17.5%). The remaining participants had a high school education or below (*n* = 46, 12.4%).

Regarding the young children’s characteristics, 206 were boys (55.4%) and 166 were girls (44.6%). Their ages ranged from 7 to 30 months, with a mean age of 18.65 months (SD = 5.77). Most children were aged 13–24 months (*n* = 254, 68.3%), followed by those aged 12 months or younger (*n* = 64, 17.2%) and those aged 25–30 months (*n* = 54, 14.5%). The term “young children” is used throughout this study to refer collectively to infants and toddlers within this age range. Detailed participant characteristics are presented in Table 1.

### 3.2. Descriptive Statistics of the Study Constructs

#### 3.2.1. Foundational Caregiving Competence

As shown in Table 2, the overall mean score for foundational caregiving competence was 3.94 (SD = 0.58). Among the three dimensions, childcare competence had the highest mean (M = 4.20, SD = 0.65), followed by child safety and protection (M = 3.87, SD = 0.66) and young child development knowledge (M = 3.74, SD = 0.68). At the item level, diaper-changing techniques had the highest mean (M = 4.37, SD = 0.72), whereas knowledge of child first aid, choking response, and wound care had the lowest mean (M = 3.55, SD = 0.85).

#### 3.2.2. Developmental Parenting and Interaction Competence

As shown in Table 3, the overall mean score for developmental parenting and interaction competence was 3.77 (SD = 0.57). Among the four dimensions, parental warmth and responsiveness had the highest mean (M = 3.91, SD = 0.68), followed by parental guidance and regulation (M = 3.83, SD = 0.71), parent–child interaction and shared learning (M = 3.68, SD = 0.75), and parental role and family management (M = 3.68, SD = 0.59). At the item level, the highest means were observed for building secure attachment with young children and preventing verbal abuse, violence, or neglect toward young children (both M = 3.96), whereas coping strategies for family stress and conflict had the lowest mean (M = 3.49, SD = 0.74).

#### 3.2.3. Eudaimonic Parental Well-Being

As shown in Table 4, the overall mean score for eudaimonic parental well-being was 4.58 (SD = 0.48). Among the three dimensions, parental sense of achievement from young children’s development had the highest mean (M = 4.69, SD = 0.43), followed by parent–child closeness (M = 4.60, SD = 0.45) and meaning and fulfillment in parenting (M = 4.47, SD = 0.61). At the item level, seeing one’s child’s growth and progress had the highest mean (M = 4.81, SD = 0.42), whereas being a parent makes life feel more beautiful had the lowest mean (M = 4.27, SD = 0.85). The high overall mean and relatively limited variability suggested that scores were concentrated toward the upper end of the scale.

Distributional characteristics were also examined for the three study constructs. Skewness values ranged from −1.130 to −0.309, and kurtosis values ranged from 0.397 to 1.199. Foundational caregiving competence and developmental parenting and interaction competence showed mild negative skewness, whereas eudaimonic parental well-being showed a more pronounced negative skewness. Nevertheless, the skewness and kurtosis values did not indicate severe univariate non-normality.

### 3.3. Correlations Among the Study Constructs

Pearson correlation analyses indicated significant positive associations among the study constructs (Table 5). Foundational caregiving competence was positively correlated with eudaimonic parental well-being (*r* = 0.31, *p* < 0.001), and the correlations between its three dimensions and eudaimonic parental well-being ranged from 0.25 to 0.31.

Developmental parenting and interaction competence was positively correlated with eudaimonic parental well-being (*r* = 0.39, *p* < 0.001). Among its dimensions, parental warmth and responsiveness had the strongest correlation with eudaimonic parental well-being (*r* = 0.38), followed by parental guidance and regulation (*r* = 0.37), parental role and family management (*r* = 0.29), and parent–child interaction and shared learning (*r* = 0.28; all *p* < 0.001).

Foundational caregiving competence was strongly correlated with developmental parenting and interaction competence (*r* = 0.79, *p* < 0.001). Correlations between their respective dimensions ranged from 0.46 to 0.69. This strong association indicated substantial overlap between the two competence constructs, whose discriminant validity was examined further in the measurement model.

### 3.4. Measurement Model Assessment

The measurement model was evaluated before the structural model was assessed. As shown in Table 6, the dimension-level indicator loadings ranged from 0.74 to 0.89. Cronbach’s α values ranged from 0.84 to 0.86, composite reliability values ranged from 0.90 to 0.91, and average variance extracted (AVE) values ranged from 0.70 to 0.78. These results supported the internal consistency and convergent validity of the measurement model.

Discriminant validity was assessed using the heterotrait–monotrait ratio of correlations (HTMT). The HTMT values ranged from 0.36 to 0.89 and remained below the commonly used threshold of 0.90. However, the HTMT value between foundational caregiving competence and developmental parenting and interaction competence was 0.89, approaching the upper threshold. The findings therefore provided acceptable but borderline support for the discriminant validity of the two parenting competence constructs, and their empirical distinction should be interpreted cautiously.

### 3.5. Structural Model and Indirect Association

**Figure 1:**
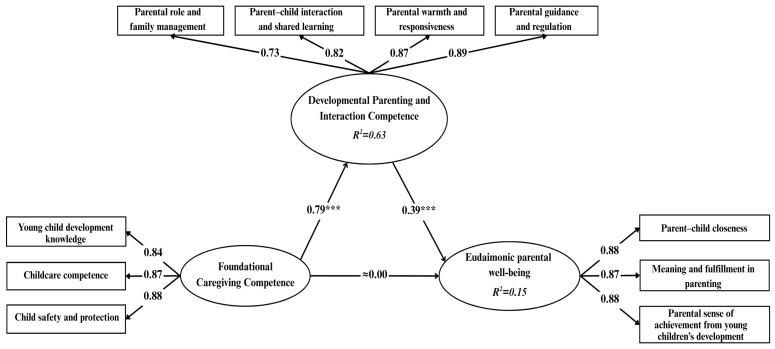
PLS-SEM structural model of the associations among foundational caregiving competence, developmental parenting and interaction competence, and eudaimonic parental well-being. Note. Standardized path coefficients are shown. Values within the endogenous constructs represent *R*^2^. The estimated paths represent statistical associations and should not be interpreted as causal or temporal relationships. *** *p* < 0.001.

Table 7 and Figure 1 present the structural model results. Foundational caregiving competence was positively associated with developmental parenting and interaction competence (β = 0.79, *t* = 39.02, *p* < 0.001), and developmental parenting and interaction competence was positively associated with eudaimonic parental well-being (β = 0.39, *t* = 5.23, *p* < 0.001), supporting H1 and H2.

The direct association between foundational caregiving competence and eudaimonic parental well-being was not significant (β = 0.00, *t* = 0.05, *p* = 0.96), whereas the indirect association through developmental parenting and interaction competence was significant (β = 0.31, *t* = 5.14, *p* < 0.001), supporting H3. The total association was also significant (β = 0.31, *t* = 6.55, *p* < 0.001).

The model explained 63% and 15% of the variance in developmental parenting and interaction competence and eudaimonic parental well-being, respectively. Effect sizes were large for foundational caregiving competence → developmental parenting and interaction competence (*f*^2^ = 1.725), small for developmental parenting and interaction competence → eudaimonic parental well-being (*f*^2^ = 0.069), and negligible for foundational caregiving competence → eudaimonic parental well-being (*f*^2^ = 0.000).

Stone–Geisser *Q*^2^ values were 0.438 and 0.114, respectively. Although all *Q*^2^ predict values were positive, PLS-SEM outperformed the linear benchmark for only three of seven indicators, indicating limited out-of-sample predictive performance. VIF values ranged from 1.52 to 2.77 and did not indicate problematic collinearity, although they could not rule out common method bias. Given the cross-sectional design, the indirect association was interpreted as an associative statistical pattern rather than causal mediation or temporal ordering.

## 4. Discussion

### 4.1. Functional Differentiation of Parenting Competence in Relation to Maternal Well-Being

The findings support a multidimensional view of parenting competence. Foundational caregiving competence was strongly associated with developmental parenting and interaction competence, but its direct association with eudaimonic parental well-being was negligible after the latter was included in the model. By contrast, developmental parenting and interaction competence was positively associated with eudaimonic parental well-being, and the indirect association was significant. Foundational caregiving competence concerns practical and protective capacities related to daily care, developmental knowledge, health, and safety, whereas developmental parenting and interaction competence encompasses emotional responsiveness, behavioral guidance, parent–child interaction, role adaptation, and family management [10,14].

This functional distinction may help interpret the absence of a significant direct association between foundational caregiving competence and maternal well-being. Practical caregiving competence facilitates the management of immediate care demands yet appears less directly linked to the specific dimensions of meaning, achievement, fulfillment, and relational satisfaction derived from parenting [21,22]. Developmental parenting and interaction competence may be more closely associated with these experiences because these competencies reflect parenting as enacted directly within parent–child and family relationships. However, these cross-sectional findings preclude the establishment of temporal or causal ordering.

The model explained only 15% of the variance in eudaimonic parental well-being, with limited effect size and out-of-sample predictive performance. Parenting competence should therefore be regarded as one of many psychosocial correlates of maternal well-being. Other unmeasured factors, including social support, partner involvement, family functioning, maternal psychological characteristics, socioeconomic resources, and work–family balance, may be associated with both perceived parenting competence and eudaimonic parental well-being. Given the cross-sectional nature of this study, these alternative explanations remain plausible, and the observed associations should not be attributed solely to the modeled parenting competence constructs.

Previous studies have linked parenting self-perceptions, competence, parental identity, and participation in parent–child programs with parental well-being, efficacy, and behavior [28,29,30,31,32,33,34,35]. The present findings provide preliminary evidence that foundational caregiving competence and developmental parenting and interaction competence are closely related but functionally distinguishable domains. They do not establish a causal mechanism or indicate that developmental parenting and interaction competence is the only relevant domain. Given the substantial empirical overlap between the two constructs, they should not be treated as entirely independent targets of family support. Parenting programs may therefore integrate practical caregiving knowledge with relational, developmental, and role-oriented components rather than addressing each competence domain separately.

### 4.2. Developmental Parenting and Interaction Competence in Relation to Eudaimonic Parental Well-Being

Developmental parenting and interaction competence was the parenting domain most closely associated with eudaimonic parental well-being. This construct encompasses emotional responsiveness, behavioral guidance, parent–child interaction and shared learning, parental role adaptation, and family management. These capacities reflect how parenting knowledge is applied within everyday relational and family contexts. The findings are consistent with a domain-specific view of parenting self-efficacy, in which perceived capability is linked to specific parenting tasks and situations rather than conceptualized as a global, generalized construct [11,12,13]. However, the present study did not compare competing theoretical models and therefore cannot establish a refinement of parenting self-efficacy theory.

Parenting education commonly emphasizes childcare knowledge, health, safety, and child development. These domains remain essential, particularly for first-time mothers. Evidence from family and parenting interventions also highlights the relevance of responsive interaction, behavioral guidance, attachment-related processes, and co-parenting [36,37,38]. The present findings are consistent with this broader perspective because developmental parenting and interaction competence includes both relational and role-oriented capacities. However, the study did not compare intervention components and cannot determine whether targeting these capacities would be more effective than focusing on foundational caregiving competence.

The transition to parenthood involves adjustments in family roles, responsibilities, and relationships [39,40]. Developmental parenting and interaction competence may be relevant within this context because it includes parental role adaptation and family management. Relationally responsive parenting has also been associated with positive adjustment, meaning making, and family relationships during early parenthood [41,42]. These findings provide a plausible context for the observed association with eudaimonic parental well-being, but they do not establish a causal process.

Related research has emphasized the importance of supporting parents as they adapt to changing family structures and caregiving roles [43]. The present results identify developmental parenting and interaction competence as a salient correlate of maternal well-being. Longitudinal or intervention-based research is needed to determine whether changes in this competence domain precede subsequent shifts in maternal well-being.

### 4.3. Implications for Women’s Mental Health and Parenting Support

The findings may inform women’s mental health support during early parenthood by suggesting that parenting education should extend beyond the transmission of childcare knowledge alone. Support programs may consider integrating foundational caregiving competence with emotional responsiveness, parental confidence, and role-related adaptation. Positive postpartum well-being is shaped by perceived support, emotional adjustment, and everyday parenting conditions [3], while evidence from Taiwan indicates that maternal confidence and social support are closely related to parenting stress among first-time mothers [8]. Accordingly, parenting support may be better aligned with the needs of first-time mothers when it addresses both practical childcare needs and the relational conditions associated with confidence, meaning, and stability in the parenting role.

This interpretation is consistent with evidence that support during the transition to parenthood should address psychological well-being, emotional adaptation, and parenting confidence in addition to technical caregiving skills [24,25,44]. Parenting education programs may therefore include complementary components related to foundational caregiving and safety, responsive parent–child interaction, behavioral guidance and communication, parenting stress management, and parental role adaptation. Such an approach reflects the distinct, yet closely related competence domains identified in this study. However, the present findings do not establish the effectiveness of any specific program structure.

The findings also support considering these components as complementary rather than arranging them as a fixed sequence of intervention steps. Health visiting and home-based support services, together with community-based programs, parenting newsletters, and parent education initiatives, may help first-time parents access timely information, receive tailored support, and normalize early parenting concerns [45,46,47,48,49,50]. These resources may be particularly valuable when integrated with broader support for maternal mental health, parent–child interaction, family role adjustment, and confidence-building [51,52]. Their effects on parenting competence and maternal well-being should nevertheless be evaluated through longitudinal or intervention-based research.

### 4.4. Cultural Context, Limitations, and Future Research

The findings should be interpreted within the sociocultural and institutional context of northern Taiwan. Participants were first-time mothers whose young children attended licensed infant daycare centers, and most had university or graduate education. Taiwanese parenting experiences are shaped by expectations concerning competent motherhood, family responsibility, and access to formal childcare [16,17,53]. Although tertiary educational attainment has increased nationally [54], the present sample had relatively strong educational and institutional resources.

Maternal well-being is shaped by family relationships, social expectations, and structural conditions [55]. Although intensive motherhood norms may increase role-related pressure [56,57], the high level of eudaimonic parental well-being observed in this study may also reflect the cultural and developmental context of early motherhood. In Taiwan, Confucian values emphasizing family continuity, parental responsibility, and the significance of motherhood may strengthen mothers’ sense of purpose, achievement, and relational fulfillment. The elevated scores may also partly reflect an early-parenthood “honeymoon phase,” supported by the novelty of motherhood, close parent–child bonding, and family support. The findings may therefore be most applicable to relatively well-educated first-time mothers with access to licensed childcare services in northern Taiwan. They should not be generalized to mothers who primarily use informal childcare arrangements, live in rural areas, or have fewer educational and socioeconomic resources. Longitudinal research is needed to determine whether these positive perceptions remain stable as parenting demands become more complex.

Several methodological limitations should be noted. The cross-sectional design precludes causal or temporal conclusions, and the single-source, self-report design may have introduced common method variance and social desirability bias. Although VIF values did not indicate problematic collinearity [26,27], they could not rule out common method bias. Procedural safeguards included anonymous and voluntary participation and the collection of no identifying information. Future studies should employ longitudinal, multi-wave designs to decouple the measurement of predictor and outcome variables. Simultaneously, leveraging multi-source data, observational protocols, or marker variables is critical to rigorously controlling for common method variance.

The measures were derived from or adapted from Taiwanese parenting and parental-joy instruments. Although their reliability and construct validity were supported in the present sample, their broader cross-cultural applicability remains uncertain. The high mean, limited variability, and negative skewness of eudaimonic parental well-being suggest a possible ceiling effect. However, neither a formal ceiling-effect analysis nor an a priori power analysis was conducted. These psychometric limitations may have reduced the measure’s sensitivity to differences among mothers with relatively high levels of well-being and may have attenuated the observed associations and explanatory power of the model.

The sample did not include mothers using informal childcare arrangements or families from rural or socioeconomically disadvantaged backgrounds [58,59,60]. Future studies should recruit participants from more diverse demographic, geographic, socioeconomic, and childcare contexts; use internationally validated measures or instruments with greater sensitivity at the upper end of the response range; and incorporate temporally separated assessments, multiple informants, behavioral observations, objective indicators, or marker variables. Longitudinal studies should assess parenting competence and eudaimonic parental well-being at multiple time points during early parenthood to examine temporal ordering and changes across developmental stages. Multi-informant studies could combine maternal self-reports with partner reports, or structured observations. Intervention-based studies using randomized or quasi-experimental designs could more rigorously examine whether changes in foundational caregiving competence or developmental parenting and interaction competence are followed by subsequent changes in maternal well-being.

## 5. Conclusions

This study examined the distinct associations of foundational caregiving competence and developmental parenting and interaction competence with eudaimonic parental well-being among first-time mothers. By distinguishing practical caregiving capacities from relationally enacted parenting competence, the study presents parenting competence as a multidimensional construct associated with eudaimonic parental well-being.

Foundational caregiving competence was strongly associated with developmental parenting and interaction competence, but its direct association with eudaimonic parental well-being was negligible and not statistically significant. Developmental parenting and interaction competence was positively associated with eudaimonic parental well-being, and the indirect association through this construct was statistically significant. These findings suggest that relational, developmental, and role-oriented parenting capacities may be more closely associated with mothers’ experiences of meaning, achievement, fulfillment, and relational satisfaction than foundational caregiving competence alone. The cross-sectional design, however, does not permit temporal or causal conclusions.

These findings advance our understanding of how distinct parenting competence domains differentially relate to maternal well-being during the critical period of early parenthood. Parenting education and family support may consider integrating childcare knowledge, health and safety information, responsive parent–child interaction, behavioral guidance, parental role adaptation, stress management, and confidence-building resources. Although the study did not evaluate a specific intervention, the identified competence domains may inform future program development and assessment.

Maternal competence should not be interpreted as an individual responsibility detached from wider family and social conditions. Parental identity, parenting behavior, maternal adaptation, and parent–child relationship quality are closely linked to parental well-being [61,62,63]. The findings indicate that foundational caregiving competence and developmental parenting and interaction competence are closely related but functionally distinguishable. Their associations with maternal eudaimonic well-being should be understood within the wider context of family relationships, childcare services, workplace conditions, and social support networks.

## Figures and Tables

**Table 1:** Demographic characteristics of participants (*N* = 372).

Variables	Category	*n*	%
Maternal education level	≤High school	46	12.4
	University	261	70.2
	Graduate school	65	17.5
Young Child’s gender	Boy	206	55.4
	Girl	166	44.6
Young Child age (months)	≤12 months	64	17.2
	13–24 months	254	68.3
	25–30 months	54	14.5
	Mean ± SD	18.65 ± 5.77	

Note. Percentages may not sum to 100.0% due to rounding.

**Table 2:** Descriptive statistics for foundational caregiving competence.

Dimension	Item	Mean	SD
Childcare competence		4.20	0.65
	Breastfeeding or breast milk expression methods and benefits.	4.25	0.84
	Preparing formula milk and burping techniques after feeding.	4.33	0.73
	Preparation of complementary foods for young children.	4.10	0.88
	Feeding techniques for complementary foods.	4.16	0.79
	Arranging young children’s sleep schedules, environments, and positions.	4.12	0.78
	Diaper-changing techniques.	4.37	0.72
	Bathing techniques for young children.	4.28	0.77
	Selecting appropriate clothing and dressing young children.	4.26	0.75
	Oral hygiene care for young children.	3.98	0.87
Young child development knowledge		3.74	0.68
	Understanding young children’s physical development milestones.	3.78	0.75
	Understanding young children’s cognitive development.	3.73	0.75
	Understanding young children’s language development.	3.73	0.76
	Understanding young children’s social development.	3.68	0.78
	Understanding young children’s emotional development.	3.59	0.78
	Understanding traditional child growth customs (e.g., full-month celebration, saliva ritual, object-grabbing ceremony).	3.87	0.93
	Understanding developmental screening tools for young children.	3.83	0.80
Child safety and protection		3.87	0.66
	Home safety inspection to prevent hazards (e.g., sharp objects, medications).	4.02	0.71
	Safety precautions when taking young children outside or traveling in vehicles.	4.05	0.71
	Knowledge of common child illnesses, prevention, and vaccination.	3.95	0.76
	Care strategies when young children are ill.	3.80	0.73
	Knowledge of child first aid, choking response, and wound care.	3.55	0.85
Overall foundational caregiving competence		3.94	0.58

Note. Responses were rated on a five-point scale ranging from 1 (fully incompetent) to 5 (fully competent).

**Table 3:** Descriptive statistics for developmental parenting and interaction competence.

Dimension	Item	Mean	SD
Parental role and family management		3.68	0.59
	Fulfilling maternal roles and responsibilities.	3.92	0.65
	Cooperative parenting with family members.	3.75	0.76
	Balancing work and family roles.	3.60	0.76
	Coping strategies for family stress and conflict.	3.49	0.74
	Family resources and financial management.	3.60	0.76
	Marital communication and relationship maintenance.	3.64	0.77
	Maintaining positive family relationships.	3.75	0.75
Parental warmth and responsiveness		3.91	0.68
	Building secure attachment with young children.	3.96	0.72
	Recognizing young children’s crying signals and responding appropriately.	3.90	0.78
	Positive responsiveness toward young children.	3.95	0.70
	Recognizing and responding to young children’s needs.	3.93	0.71
	Understanding separation anxiety and soothing techniques.	3.78	0.80
Parent–child interaction and shared learning		3.68	0.75
	Knowledge and skills for parent–child play activities.	3.75	0.80
	Techniques for parent–child shared reading or learning.	3.64	0.81
	Strategies for family meals with young children.	3.67	0.83
	Creating a supportive home learning environment for young children.	3.64	0.83
Parental guidance and regulation		3.83	0.71
	Establishing consistent daily routines.	3.87	0.78
	Encouraging socially acceptable behaviors.	3.76	0.80
	Preventing and limiting dangerous behaviors.	3.88	0.80
	Managing problematic child behaviors (e.g., biting, aggression).	3.68	0.87
	Preventing verbal abuse, violence, or neglect toward young children.	3.96	0.79
Overall developmental parenting and interaction competence		3.77	0.57

Note. Responses were rated on a five-point scale ranging from 1 (fully incompetent) to 5 (fully competent).

**Table 4:** Descriptive statistics for eudaimonic parental well-being.

Dimension	Item	Mean	SD
Meaning and fulfillment in parenting		4.47	0.61
	Being a parent gives me goals in life.	4.52	0.71
	Being a parent allows me to transmit my core life values to my child.	4.39	0.81
	Parenthood helps me experience the completeness of life.	4.52	0.77
	Experiencing life together with my child.	4.67	0.54
	My child makes me feel hopeful about the future.	4.42	0.78
	Parenthood gives me a sense of self-fulfillment.	4.31	0.86
	Being a parent gives me deeper insights into life.	4.68	0.54
	Being a parent makes life feel more beautiful.	4.27	0.85
Parental sense of achievement from young children’s development		4.69	0.43
	Seeing my child’s growth and progress.	4.81	0.42
	My child shares what they have learned with me.	4.59	0.68
	Watching my child’s learning ability improve.	4.80	0.42
	My child enjoys learning new things.	4.66	0.54
	My child’s performance makes me feel proud.	4.61	0.58
Parent–child closeness		4.60	0.45
	My child makes me feel cared for and loved.	4.33	0.76
	I feel that I am important to my child.	4.65	0.56
	Hearing my child say “Mom, I love you”.	4.28	1.03
	My child seeks affection from me.	4.75	0.46
	My child initiates hugging me.	4.76	0.48
	The intimate relationship with my child makes me feel satisfied.	4.73	0.51
	Interacting with my child makes me feel happy.	4.68	0.56
Overall eudaimonic parental well-being		4.58	0.48

Note. Responses were rated on a five-point scale ranging from 1 (strongly disagree) to 5 (strongly agree).

**Table 5:** Pearson correlations among the study constructs and dimensions.

Variable	1	2	3	4	5	6	7	8	9	10	11	12	13
**1. Foundational caregiving competence**	1												
2. Childcare competence	0.88 ***	1											
3. Young child development knowledge	0.86 ***	0.62 ***	1										
4. Child safety and protection	0.87 ***	0.68 ***	0.60 ***	1									
**5. Developmental parenting and interaction competence**	0.79 ***	0.66 ***	0.67 ***	0.74 ***	1								
6. Parental role and family management	0.55 ***	0.46 ***	0.51 ***	0.48 ***	0.74 ***	1							
7. Parental warmth and responsiveness	0.73 ***	0.62 ***	0.61 ***	0.68 ***	0.86 ***	0.54 ***	1						
8. Parent–child interaction and shared learning	0.63 ***	0.51 ***	0.54 ***	0.61 ***	0.84 ***	0.45 ***	0.62 ***	1					
9. Parental guidance and regulation	0.72 ***	0.61 ***	0.59 ***	0.69 ***	0.89 ***	0.55 ***	0.73 ***	0.69 ***	1				
**10. Eudaimonic parental well-being**	0.31 ***	0.26 ***	0.25 ***	0.31 ***	0.39 ***	0.29 ***	0.38 ***	0.28 ***	0.37 ***	1			
11. Meaning and fulfillment in parenting	0.32 ***	0.26 ***	0.27 ***	0.31 ***	0.39 ***	0.29 ***	0.38 ***	0.29 ***	0.33 ***	0.89 ***	1		
12. Parental sense of achievement from young children’s development	0.23 ***	0.21 ***	0.19 ***	0.21 ***	0.29 ***	0.23 ***	0.28 ***	0.17 ***	0.30 ***	0.88 ***	0.65 ***	1	
13. Parent–child closeness	0.26 ***	0.22 ***	0.18 ***	0.28 ***	0.34 ***	0.23 ***	0.31 ***	0.26 ***	0.35 ***	0.87 ***	0.62 ***	0.74 ***	1

Note. *N* = 372. Values are Pearson correlation coefficients. *** *p* < 0.001.

**Table 6:** Measurement model assessment.

Construct/Indicator	Loading	Cronbach’s α	CR	AVE	HTMT
Foundational caregiving competence	—	0.84	0.90	0.76	DPIC = 0.89; EPWB = 0.36
Childcare competence	0.88	—	—	—	—
Young child development knowledge	0.85	—	—	—	—
Child safety and protection	0.89	—	—	—	—
Developmental parenting and interaction competence	—	0.86	0.91	0.78	EPWB = 0.45
Parental role and family management	0.74	—	—	—	—
Parental warmth and responsiveness	0.88	—	—	—	—
Parent–child interaction and shared learning	0.83	—	—	—	—
Parental guidance and regulation	0.74	—	—	—	—
Eudaimonic parental well-being	—	0.86	0.90	0.70	—
Meaning and fulfillment in parenting	0.87	—	—	—	—
Parental sense of achievement from young children’s development	0.88	—	—	—	—
Parent–child closeness	0.89	—	—	—	—

Note. DPIC = developmental parenting and interaction competence; EPWB = eudaimonic parental well-being; CR = composite reliability; AVE = average variance extracted; HTMT = heterotrait–monotrait ratio of correlations. Dimension-level loadings are reported for the higher-order constructs, whereas Cronbach’s α, CR, and AVE are based on the measurement specifications used in the SmartPLS model. Dashes indicate not applicable.

**Table 7:** Structural model and hypothesis testing.

Hypothesis/Association	β	*t*	*p*	Result
H1: FCC → DPIC	0.79	39.02	<0.001	Supported
H2: DPIC → EPWB	0.39	5.23	<0.001	Supported
FCC → EPWB	0.00	0.05	0.96	Not significant
H3: FCC → DPIC → EPWB	0.31	5.14	<0.001	Supported
Total association: FCC → EPWB	0.31	6.55	<0.001	—

Note. FCC = foundational caregiving competence; DPIC = developmental parenting and interaction competence; EPWB = eudaimonic parental well-being; β = standardized path coefficient; → = hypothesized association/path between constructs. Statistical significance was assessed using 5000 bootstrap resamples. The model explained 63% of the variance in DPIC (*R*^2^ = 0.63) and 15% of the variance in EPWB (*R*^2^ = 0.15). VIF values ranged from 1.52 to 2.77.

## Data Availability

Due to ethical considerations and restrictions related to participant confidentiality, the data presented in this study are not publicly available.
